# Preservation of 5-Hydroxymethylcytosine Levels in *LRIG1* across Genomic DNA and Cell-Free DNA in Glioma Patients

**DOI:** 10.3390/genes15050535

**Published:** 2024-04-24

**Authors:** Daša Jevšinek Skok, Luka Bolha, Nina Hauptman

**Affiliations:** 1 Agricultural Institute of Slovenia, Hacquetova ulica 17, SI-1000 Ljubljana, Slovenia; dasa.jevsinekskok@kis.si; 2 Institute of Pathology, Faculty of Medicine, University of Ljubljana, Korytkova 2, SI-1000 Ljubljana, Slovenia; luka.bolha@mf.uni-lj.si

**Keywords:** cell-free DNA, glioma, 5-hydroxymethylcytosine, biomarkers

## Abstract

Cell-free DNA (cfDNA) has recently emerged as a promising minimally invasive diagnostic biomarker for various cancers. In this study, our aim was to identify cfDNA biomarkers by investigating genes that displayed significant differences between glioma patients and their corresponding controls. To accomplish this, we utilized publicly available data from the Gene Expression Omnibus, focusing on 5-hydroxymethylcytosine (5hmC) profiles in both cfDNA and genomic DNA (gDNA) from glioma patients and healthy individuals. The intersection of gene lists derived from these comparative analyses unveiled *LRIG1* and *ZNF703* as the two genes with elevated 5hmC levels in both the cfDNA of glioma patients and gDNA of glioma tissue compared to their respective controls. The gene expression data revealed both genes were upregulated in glioma tissue compared to normal brain tissue. Integration of 5hmC data revealed a strong positive correlation in the glioma tissue group between 5hmC and the gene expression of the *LRIG1* gene. Furthermore, exploration using the AmiCa web tool indicated that *LRIG1* gene expression was elevated compared to 17 other cancers included in the database, emphasizing its potential as a distinctive biomarker across multiple cancer types.

## 1. Introduction

Transcriptional regulation involves a network of interconnected complex mechanisms, with epigenetic modifications standing out as a pivotal mechanism for controlling gene expression. Among these, DNA methylation and histone modification are particularly crucial. DNA methylation plays a central role in various biological processes such as embryonic development, genomic imprinting, X-chromosome inactivation, cellular differentiation, and cancer development. This process involves the addition of a methyl group to the C5 position of cytosine, catalyzed by DNA methyltransferases, leading to the formation of 5-methylcytosine (5mC). The reverse process, demethylation, is facilitated by the ten-eleven translocation (TET) family of cytosine oxygenases. Demethylation involves the oxidation of 5mC to the intermediate state 5-hydroxymethylcytosine (5hmC). While the presence of 5hmC indicates demethylation during developmental stages, it also serves as a stable epigenetic modification in epigenetic reprogramming. Extensive research has demonstrated the detection of 5hmC in various tissues and cell types [1,2,3].

Glioma is one of the most life-threatening primary tumors, constituting approximately 80% of all cerebral tumors and represents a major cause of morbidity and mortality in neurosurgical patients. Upon diagnosis, over 90% of patients face a survival prognosis of less than 5 years [4]. Despite advancements in glioma treatment and diagnosis, its incidence and mortality rate persist at elevated levels. The pathogenesis of glioma is intricately linked to multiple factors encompassing biology, genetics, chemistry, physics, and the environment [5]. Contrary to other cancers, gliomas rarely metastasize; instead, they invade the surrounding normal brain tissue and have a high resistance to therapy. In spite of achievements made in the therapeutic field, the overall survival of glioma patients is poor [6].

Gliomas, categorized as incurable brain cancers with poor prognosis, exhibit a distinctive feature—epigenetic dysregulation. One noteworthy aspect of this dysregulation is the altered presence of 5hmC, an intermediate in the demethylation of 5mC [7]. In the context of gliomas, the screening of 5hmC, a demethylation marker resulting from 5mC oxidation, in liquid biopsy specimens represents a great potential. This approach offers opportunities for the early detection, diagnosis, prognosis, and monitoring of dynamic changes and treatment outcomes in gliomas [8].

In recent years, the advancement of high-throughput sequencing technologies has significantly enhanced the sensitivity and precision of liquid biopsy methods. These modern techniques not only facilitate the early detection of various cancers, but also serve as noninvasive means for assessing identified diseases. This versatility is particularly beneficial for suspected gliomas as it allows for a more comprehensive understanding of intracranial lesions, aiding in the tailoring of surgical strategies. Moreover, the adoption of such techniques can help to avoid unnecessary surgeries, thereby mitigating risks and reducing complications for patients [9].

In this study, our primary focus was on identifying genes with a positive difference in 5hmC levels in both the cell-free DNA (cfDNA) and genomic DNA (gDNA) of glioma patients in comparison to their corresponding normal samples. Additionally, our objective extended to investigating the relationship between 5hmC and gene expression in glioma tissue samples. We sought to discern whether the gene expression of selected genes differs among gliomas and other cancer types.

## 2. Materials and Methods

We performed an in silico analysis using publicly available data from the Gene Expression Omnibus (GEO). These data, obtained by next generation sequencing (NGS), focused specifically on 5-hydroxymethylcytosine (5hmC) in circulating cfDNA from blood samples and gDNA from tissue samples. The analyzed samples were from patients diagnosed with glioma and healthy individuals. The samples’ 5hmC data were obtained using the 5hmC-Seal method, a highly sensitive and specific technique for the detection and enrichment of DNA containing 5hmC, which is particularly valuable for research that requires the precise mapping of 5hmC throughout the genome or at specific loci. It utilizes a glucosylation reaction that targets the hydroxymethyl group of 5hmC, where a modified glucose molecule with an azide group is attached to 5hmC by the T4-β-glucosyltransferase. Subsequently, the azide enables the covalent attachment of a biotin tag through a click chemistry reaction that allows for the efficient enrichment of 5hmC-containing DNA fragments using streptavidin beads [3,8].

Our analysis included two projects, both performed on the Illumina NextSeq 500 platform (accessed 15 January 2024), which is a high-throughput NGS technology. The first project, GSE132118 [3], provided 5hmC data from cfDNA samples. It included samples from 111 individuals diagnosed with glioma and an equal number of samples from healthy volunteers serving as controls.

The second project, GSE196533 [8], provided 5hmC data from gDNA derived from tissue samples; 61 samples from glioma patients and nine samples from normal brain tissue. Additionally, this dataset included gene expression data generated using the Illumina HiSeq 4000 platform, performed on the same 61 glioma tissue samples and nine normal brain tissue samples, enabling a comprehensive genetic analysis by correlating epigenetic modifications with changes in gene expression.

To compare the selected groups, we used the limma package (version 3.57.4) in R, a statistical tool widely used in bioinformatics for analyzing data [10]. Our primary goal was to perform pairwise comparisons to identify differences in the 5hmC levels: (i) between cfDNA from glioma patients and healthy individuals, and (ii) between gDNA from glioma tissues and normal brain tissues. The false discovery rate (FDR) was determined for each gene to adjust for multiple hypothesis testing, focusing only on genes with adjusted *p*-values of less than 0.05, ensuring that the findings were statistically significant.

After identifying genes with significant differences in 5hmC levels, we applied a filter to identify genes with a positive difference in levels of 5hmC: (i) between cfDNA from glioma patients versus healthy individuals, and (ii) between gDNA from glioma versus normal brain tissues. The intersection of these lists provided two genes of interest. We further analyzed these two genes to explore their expression levels in the GSE196533 dataset using *t*-tests and assessed correlations between the 5hmC levels and gene expression in both groups, glioma and normal.

Obtained results were then used for further analysis on the expression of these two genes in different types of cancer. For this analysis, we used the web tool AmiCa (https://amica.omics.si/, accessed 18 January 2024), a web platform for studying miRNA and gene expression in different types of cancer.

Furthermore, we used the geom_boxplot function from the ggplot2 library (version 3.4.2) [11] in the R programming language (version 4.3.0) to make a graphical presentation of 5hmC levels of intersected genes. Data were presented as boxplots, which include the minimum, first quartile (25th percentile), median, third quartile (75th percentile), and maximum, which are all essential statistical values.

## 3. Results

### 3.1. 5hmC Profiles in cfDNA and gDNA of Glioma Patients

Our study included data from two different projects on the Illumina NextSeq 500 platform. In the first dataset, GSE132118, we analyzed cfDNA samples from 111 glioma patients and an equal number of healthy individuals. The second dataset, GSE196533, included gDNA from 61 glioma tissue samples and nine corresponding normal brain tissue samples, along with the gene expression data from the same samples obtained using the Illumina HiSeq 4000 platform.

**Figure 1:**
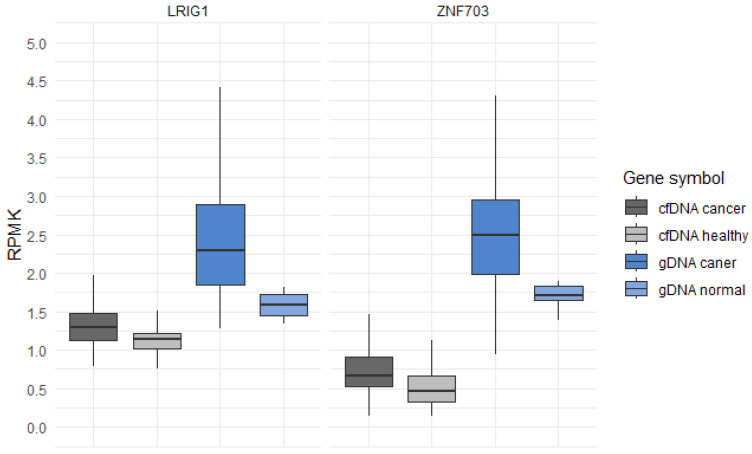
Levels of 5hmC in genes *LRIG1* and *ZNF703* in cfDNA from glioma patients compared to the cfDNA from healthy individuals, and in the gDNA of glioma tissue samples compared to the gDNA of normal tissue samples. The boxplots illustrate the distribution of a continuous variable, providing a visual representation of five summary statistics including the median, two hinges, and two whiskers. RPKM, reads per kilobase per million.

Pairwise comparisons within projects GSE132118 and GSE196533, aimed to identify differentially expressed genes, revealed a total of 7919 genes from the cfDNA comparisons and 2885 genes from the gDNA comparisons. By setting a log change in the 5hmC level (loghmC) threshold to 0.80 or higher for gDNA comparisons, we identified 121 genes with significantly different levels of 5hmC between the glioma and normal brain tissues. For cfDNA comparisons, setting a loghmC threshold to 0.15 or higher resulted in 63 significant genes (Supplementary Table S1). The intersection of gene lists that showed positive differences in 5hmC levels between glioma and normal/healthy samples resulted in two promising candidate genes: leucine-rich repeats and immunoglobulin-like domains protein 1 (*LRIG1*) and zinc finger protein 703 (*ZNF703*). Both genes exhibited substantial differences in the 5hmC levels between the groups, placing them in the top third of our list of genes, ordered according by *p*-values (Supplementary Table S1). Visual representation of the 5hmC levels of *LRIG1* and *ZNF703* is presented in Figure 1, where we utilized reads per kilobase per million (RPKM) to illustrate the distribution of 5hmC in both the cfDNA from glioma patients and gDNA from glioma tissues compared to their respective controls.

### 3.2. Interrelation between 5hmC Levels and Gene Expression in Glioma Patients

Notably, the project GSE196533, which contained data on the 5hmC levels in the tissue samples, also provided gene expression data for the same set of samples. Therefore, we could examine the expression of genes *LRIG1* and *ZNF703* in the glioma and normal tissue samples. We revealed that the expression of both genes was higher in the glioma samples compared to the normal tissues. Specifically, gene *LRIG1* had an average mean log fold-change (logFC) of 5.07 in the glioma samples and 1.27 in the normal samples, with a significant difference (*p*-value 2.02 × 10^−11^). The correlation among 5hmC status and gene expression was −0.04 (*p*-value 0.924) in the normal samples and 0.60 (*p*-value 3.68 × 10^−7^) in the glioma samples. The gene *ZNF703* exhibited the average mean logFC of 1.95 in the glioma samples and 1.61 in the normal samples, without significant difference (*p*-value 0.10). The correlation between the 5hmC levels and gene expression was 0.39 (*p*-value 0.31) in the normal samples and 0.01 (*p*-value 0.95) in the tumor samples.

We further explored the expression of *LRIG1*, which reached statistical significance in contrast to *ZNF703*, which did not. For this purpose, we used the web platform AmiCa (accessed 18 January 2024), where information on gene and miRNA expression in different cancer types can be found. The calculations in AmiCa are based on data from The Cancer Genome Atlas (TCGA). The tool allows for comparisons of gene (and miRNA) expressions between individual cancer types. Thus, using the “miRNA/gene expression” module (https://amica.omics.si/by_diseases_MTI.php, accessed 18 January 2024), searching by gene name *LRIG1*, the result showed the *LRIG1* gene to be highly expressed in glioma (TCGA project name: brain lower grade glioma, LGG) tissue compared to other cancers.

**Figure 2:**
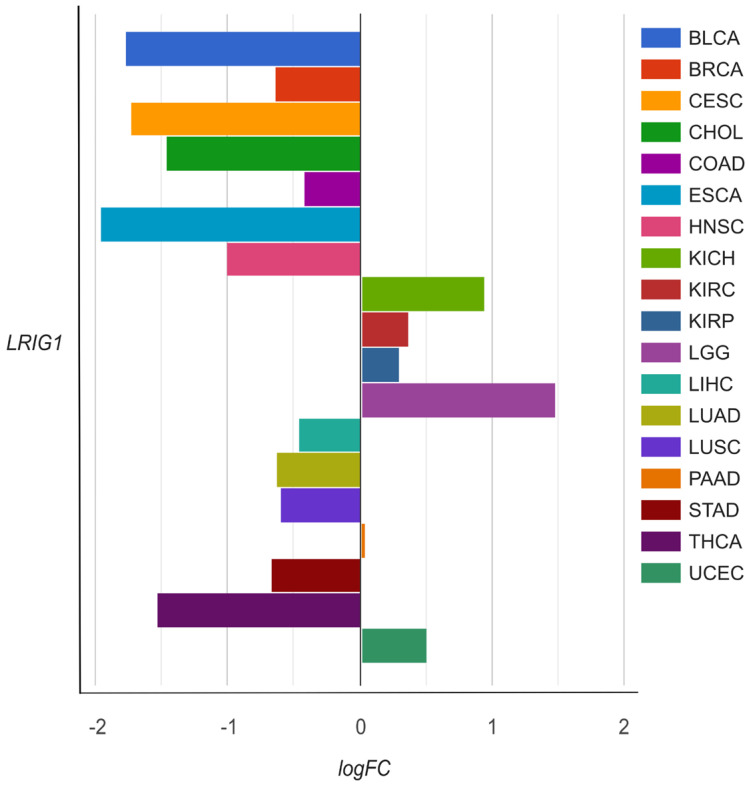
Expression of *LRIG1* gene across different cancers as presented in the AmiCa database (https://amica.omics.si/, accessed 18 January 2024). BLCA, bladder urothelial carcinoma; BRCA, breast invasive carcinoma; CESC, cervical and endocervical cancers; CHOL, cholangiocarcinoma; COAD, colon adenocarcinoma; ESCA, esophageal carcinoma; HNSC, head and neck squamous cell carcinoma; KICH, kidney chromophobe; KIRC, kidney renal clear cell carcinoma; KIRP, kidney renal papillary cell carcinoma; LGG, brain lower grade glioma; LIHC, liver hepatocellular carcinoma; LUAD, lung adenocarcinoma; LUSC, lung squamous cell carcinoma; PAAD, pancreatic adenocarcinoma; STAD, stomach adenocarcinoma; THCA, thyroid carcinoma; UCEC, uterine corpus endometrial carcinoma.

The chart shows that the *LRIG1* gene is most expressed in LGG (logFC = 1.46), followed by the kidney chromophobe (KICH) with a logFC of only 0.94 (Figure 2). Moreover, the *LRIG1* gene was shown to be downregulated in 12 cancers. This expanded analysis not only underscores the complexities of epigenetic and gene expression dynamics in glioma, but also highlights potential diagnostic and therapeutic targets, paving the way for more personalized and effective cancer treatments.

## 4. Discussion

In this investigation, we conducted an analysis utilizing available data on the 5hmC levels in cfDNA from glioma patients and healthy individuals as well as data on the 5hmC levels in gDNA from glioma tissues and normal brain tissues sourced from the GEO projects. Through pairwise group comparisons and a subsequent intersection of gene lists, we identified two genes, *LRIG1* and *ZNF703*, exhibiting higher 5hmC levels in glioma patients/tissues when compared to healthy individuals/normal brain tissue.

Furthermore, we leveraged the gene expression data available for glioma and normal brain tissues from the same patients for whom 5hmC data were accessible. This allowed us to investigate whether gene expressions differed between the glioma tissues and normal brain tissues. Notably, gene *LRIG1* demonstrated a significant upregulation in the glioma tissue compared to normal brain tissue. Additionally, we calculated the correlation between 5hmC and gene expression, revealing a strong significant positive correlation for the *LRIG1* gene.

The *LRIG* gene family was discovered when searching for paralogous genes in the human genome, based on research previously performed on Drosophila and mice, where it was discovered that this gene was regulated downstream by epidermal growth factor receptor (EGFR) [12,13]. The gene encoded a transmembrane protein containing a cell adhesion molecule with leucine-rich repeats and immunoglobulin-like domain. In Drosophila, it was established that this protein is produced by epidermal growth factor (EGF) stimulation, where it binds to EGFR, which, in turn regulates its activity through a negative feedback loop [12]. The search for a paralogous gene in humans led to the discovery of the *LRIG* gene family, namely genes *LRIG1*, *LRIG2*, and *LRIG3* [14,15,16]. The LRIG proteins are differentially expressed in human tissues, so are therefore important in different cell types [15]. Differential expression of LRIG proteins was also found in different cancers including glioma [6].

Previous studies associated LRIG1 with brain cancer [6,17,18,19], where it inhibits EGFR expression in glioblastoma cells. Moreover, gene *LRIG1* has a crucial role in some other cancer types such as bladder cancer, colorectal cancer, breast cancer, and lung cancer [20,21,22,23,24]. All of these studies proved that increased expression of LRIG1 worsens the prognosis of cancer.

In the brain, it was shown that LRIG1 inhibits EGFR expression in glioblastoma cells by causing the activation of downstream signaling pathways. EGFR, known for its significance in tumor proliferation and viability, is effectively controlled by LRIG1. Furthermore, LRIG1 plays a crucial role in influencing the expression of Bcl-2, an anti-apoptotic protein, and Topo-2, essential for maintaining DNA stability, replication, and repair processes. The impact of LRIG1 extends to curtailing EGFR expression and its signaling cascade. Simultaneously, LRIG1 modulates the Bcl-2 and Topo-2 levels, rendering glioma cells more responsive to temozolomide (TMZ) treatment. These findings emphasize LRIG1 as a promising candidate for enhancing the efficacy of TMZ utilization in glioma therapy [17].

The downregulation of EGFR caused by LRIG1 triggers cellular apoptosis and inhibits cell growth. This is achieved by inhibiting the phosphorylation of EGFR and downstream signaling pathways such as MAPK and AKT. From a therapeutic perspective, restoring LRIG1 levels in tumors, particularly gliomas, has emerged as a promising strategy for suppressing receptor-positive tumors. This suggests that manipulating LRIG1 expression may offer a novel therapeutic approach for cancers driven by the aberrant EGFR signaling. Overall, these findings position LRIG1 as a negative feedback attenuator of signaling pathways mediated by receptor tyrosine kinases, highlighting its potential importance in controlling cell fate and behavior, especially in the context of cancer [6].

Xie et al. have also shown that LRIG1 functions as a tumor suppressor in the pathogenesis of glioma via EGFR/Akt/c-Myc activation by using RNA interference (RNAi) that may effectively downregulate *LRIG1* gene expression [18]. The study conducted by Liu et al. indicates that resveratrol possesses the ability to impede the growth and proliferation of glioma while facilitating its apoptosis. This inhibitory effect is achieved through the upregulation of *LRIG1* gene expression, showcasing LRIG1 as a pivotal factor in the anti-glioma properties of resveratrol, which unveils LRIG1 as a novel biological target of resveratrol in the context of antagonizing cell proliferation and growth in glioma cells [19].

Overall, this study confronts several limitations that significantly impact the breadth and applicability of our findings. A primary constraint is the absence of data on 5mC and unmethylated DNA status, alongside 5hmC, from the same glioma patients and healthy individuals. Despite exhaustive searches within the GEO database, we were unable to identify any datasets that provided comprehensive methylation profiles—5mC, 5hmC, and unmethylated DNA—from the same cohort of glioma patients and their corresponding controls. This gap in data availability limited our ability to conduct a more holistic analysis that might reveal intricate relationships between these epigenetic modifications and glioma pathogenesis.

Moreover, setting an appropriate threshold for significant gene hits proved challenging. Optimal threshold settings were difficult to establish due to the subtle differences in 5hmC levels observed in the utilized data. Setting the threshold too high resulted in few or no relevant genes for further analysis, while a lower threshold produced an overwhelming number of hits, crucially impairing the analysis and interpretation. We empirically set the thresholds at 0.80 for gDNA and 0.15 for cfDNA, allowing us to balance the sensitivity and specificity effectively.

These constraints underscore the challenges inherent in studying complex diseases like glioma through public databases and highlight the need for more comprehensive, multi-dimensional datasets that can support the nuanced analysis required to fully understand cancer pathogenesis. We recognize the potential value of integrating diverse types of data from consistent sources and suggest that future studies may benefit from designing experiments that can overcome these limitations.

## 5. Conclusions

In our current study, we identified genes *LRIG1* and *ZNF703* with 5hmC gene levels higher in both the cfDNA and gDNA of glioma patients. Interestingly gene *LRIG1* also exhibited a strong positive correlation with gene expression data in glioma patients. Furthermore, the comparison of gene expression across different cancers showed the highest upregulation of *LRIG1* in glioma.

Moreover, our study contributes to the growing body of evidence associating LRIG1 with glioma pathogenesis, corroborating previous research highlighting LRIG1’s inhibitory effects on EGFR expression and downstream signaling pathways. These findings reinforce LRIG1’s significance and present opportunities for tailored therapeutic interventions.

In the broader context of glioma diagnosis and treatment, our exploration of the 5hmC levels in liquid biopsy specimens showcases its potential for the early detection, diagnosis, prognosis, and dynamic monitoring of treatment outcomes. The application of high-throughput sequencing technologies, as demonstrated in our study, enhances the precision and sensitivity of liquid biopsy methods, presenting a noninvasive avenue for comprehensive assessment and tailored surgical strategies in suspected glioma cases.

## Data Availability

All data used in this paper are available in the article.

## Supplementary Materials

The following supporting information can be downloaded at: https://www.mdpi.com/article/10.3390/genes15050535/s1. Table S1: Genes with statistical difference in 5hmC levels between glioma and normal samples.
